# Optimal fractionation and timing of weekly cone-beam CT in daily surface-guided radiotherapy for breast cancer

**DOI:** 10.1186/s13014-023-02279-4

**Published:** 2023-07-05

**Authors:** Haiyan Peng, Han Yang, Jinyan Lei, Xinyao Dai, Panpan Cao, Fu Jin, Huanli Luo

**Affiliations:** Departments of Radiation Oncology, Chongqing University Cancer Hospital, Chongqing, People’s Republic of China

**Keywords:** Postmastectomy radiotherapy, Multiple “SGRT + CBCT” modes, Patient-specific parameters, Setup error, Dose distribution

## Abstract

**Purpose:**

Surface-guided radiotherapy (SGRT) has been demonstrated to be a promising supplement to cone-beam computed tomography (CBCT) in adjuvant breast cancer radiotherapy, but a rational combination mode is lacking in clinical practice. The aim of this study was to explore this mode and investigate its impact on the setup and dose accuracy.

**Methods and materials:**

Daily SGRT and weekly CBCT images were acquired for 23 patients with breast cancer who received conventional fractionated radiotherapy after lumpectomy. Sixteen modes were acquired by randomly selecting one (CBCT_1_), two (CBCT_ij_), three (CBCT_ijk_), four (CBCT_ijkl_), and five (CBCT_12345_) images from the CBCT images for fusion with the SGRT. The CTV-PTV margins, OAR doses, and dose coverage (V95%) of PTV and CTV was calculated based on SGRT setup errors with different regions of interest (ROIs). Dose correlations between these modalities were investigated using Pearson and Spearman’s methods. Patient-specific parameters were recorded to assess their impact on dose.

**Results:**

The CTV-PTV margins decreased with increasing CBCT frequencies and were close to 5 mm for CBCT_ijkl_ and CBCT_12345_. For the ipsilateral breast ROI, SGRT errors were larger in the AP direction, and target doses were higher in all modes than in the whole breast ROI (P < 0.05). In the ipsilateral ROI, the target dose correlations between all modes increased with increasing CBCT time intervals, decreased, and then increased with increasing CBCT frequencies, with the inflection point being CBCT participation at week 5. The dose deviations in CBCT_123_, CBCT_124_, CBCT_125_, CBCT_ijkl_, and CBCT_12345_ were minimal and did not differ significantly (P > 0.05). There was excellent agreement between CBCT_124_ and CBCT_1234_, and between (CBCT_ijkl_, CBCT_12345_) and CBCT_125_ in determining the classification for the percentage of PTV deviation (Kappa = 0.704–0.901). In addition, there were weak correlations between the patient’s D_ips_b_ (ipsilateral breast diameter with bolus) and CTV doses in modes with CBCT participation at week 4 (R = 0.270 to 0.480).

**Conclusions:**

Based on weekly CBCT, these modes with ipsilateral ROI and a combination of daily SGRT and a CBCT frequency of ≥ 3 were recommended, and CBCT was required at weeks 1 and 2 for CBCT_ijk_.

**Supplementary Information:**

The online version contains supplementary material available at 10.1186/s13014-023-02279-4.

## Introduction

Radiation therapy (RT) plays an important role in improving the survival rate of patients with breast cancer [1], and accurate tumour localisation during RT is critical for tumour control and toxicity in healthy tissues [2]. However, localisation is susceptible to patient setup errors, breast morphological changes, respiration, etc [3]. Given these factors, image-guided radiation therapy (IGRT) has been introduced into clinical practice [4]. Cone-beam computed tomography (CBCT), the gold standard for IGRT, can provide three-dimensional (3D) anatomical images, which can be registered to the planning CT images to check tumour information. Nevertheless, CBCT has the following drawbacks: additional dose, time consumption, and inability to monitor setup errors. Therefore, weekly CBCT scans are routinely utilised as a conventional image-guided modality in breast cancer RT [5].

Surface-guided radiotherapy (SGRT), a complementary approach to CBCT, has been developed in recent years [6]. It is a relatively recent technology that generates 3D surfaces in various ways, such as structured light, stereo vision, time of flight, or laser scanning. SGRT uses one or more camera units to obtain real-time images for positioning, motion monitoring, and respiratory gating [7]. A substantial and growing body of research has investigated the use of SGRT during breast cancer RT in free-breathing (FB) and deep-inspiration breath-holding (DIBH) conditions [8–13]. For patients undergoing FB, the mean systematic and random setup errors using SGRT were decreased to 0.8–2.9 mm and 0.4–1.8 mm, respectively [9]. For patients undergoing DIBH, the median standard deviation of the breath-hold level could be as low as 0.3 mm [13]. These studies highlight the powerful capabilities of SGRT in breast cancer RT. However, SGRT cannot acquire in vivo anatomical images, which can be gathered via CBCT [14].

Is there a method to combine SGRT and CBCT to guide breast cancer RT more accurately? This has been tested in several previous studies. Sauer recently proposed an SGRT-only protocol after the first five RT fractions and any necessary CBCT correction [11]. Zhao created a new workflow in which both SGRT and CBCT were used in the first three RT fractions, followed by daily SGRT and weekly CBCT [15]. In addition, there are numerous other “SGRT + CBCT” modalities that are being applied in clinical practice, such as daily SGRT and biweekly CBCT, but these modalities vary greatly among different centres due to the absence of guidelines, especially for breast cancer RT.

Therefore, this study examined 16 “SGRT + CBCT” modalities according to the frequency and timing of CBCT scanning and tried to get the optimal modality for breast cancer RT through evaluating setup errors and dosimetric differences. Meanwhile, the impact of patient-specific parameters, such as age and body mass index (BMI), on doses in the target volume and organs at risk (OARs) were assessed in different modalities. The dependence of the regions of interest (ROIs) on these modalities was also investigated to evaluate the clinical differences in various oncologic centres.

## Materials and methods

### Patient data

Twenty-three patients with breast cancer who received conventional fractionated radiotherapy after lumpectomy between January 2021 and March 2022 were included in this study. All patients provided written informed consent and the study was approved by the ethics committee of our hospital. The cohort consisted of 11 left- and 12 right-sided patients with breast cancer. They were planned and treated on a thoraxboard (HipFix, CIVCO Medical Solutions, IA, USA) with their arms above their head, and a cushion stuffed with foam pieces (FuRui, China) was integrated to improve comfort and positioning accuracy. A big-bore CT scanner (Brilliance, Cleveland, OH) was used to perform a FB scan from the mandible to the lower abdomen with a 5 mm slice thickness. The tube current was 280 mA and the tube voltage was 120 kV. All patients received postoperative RT in the FB condition on a Varian iX linear accelerator (Varian Medical Systems, Palo Alto, CA, USA) equipped with an on-board imager and a SGRT system.

### Catalyst HD system

In this study, Catalyst HD (C-Rad, Upsalla, Sweden) was used for SGRT [16]. It uses three cameras mounted on the ceiling of the treatment room and employs optical triangulation to obtain the patient surface information. The camera resolution was 640 × 480 pixels, and the maximum scan range was 1.1 × 1.4 × 2.4 m^3^. This optical system compares the actual surface with the reference surface from the planning CT or from a reference surface acquired during treatment and obtains setup errors using a non-rigid algorithm with optimal gains and integral times [17]. Before the clinical use of Catalyst HD, daily checks were performed, and thermal equilibrium and system drift were considered.

### RT planning

Planning CT images were imported into a treatment planning system (TPS) (Eclipse 15.6, Varian Medical Systems Inc., Palo Alto, CA, USA). The clinical target volume (CTV) and OARs were contoured by radiation oncologists following the guidelines and recommendations of the Radiation Therapy Oncology Group (RTOG) 1304 protocol [18]. The CTV included the chest wall (CW), internal lymph mammary nodes (IMNs), and axillary and supraclavicular lymph nodes, while the OARs included the heart, lungs, and contralateral breast. A 5-mm margin was added to the CTV to define PTV.

For all cases, the prescribed dose was 50 Gy in 25 fractions with 6 MV photons, and intensity-modulated radiotherapy (IMRT) was used with a dose rate of 400 MU/min and a calculation grid of 0.25 cm. The IMRT plan generally consisted of eight fields. Two V-shaped fields (20° and 340°) covered the supraclavicular region, and three pairs of tangential fields covered the supraclavicular region and CW (45°, 52°, 60°, 230°, 238°, and 246° for right-sided breast cancer; and 115°, 125°, 135°, 305°, 310°, and 320° for left-sided breast cancer). When the internal breast region was irradiated, an extra field (20°) was applied. In all plans, the prescribed dose covered at least 95% of the PTV, and dose-volume constraints for OARs were adopted from the RTOG-1304 protocol.

### Image guidance workflow

**Fig. 1:**
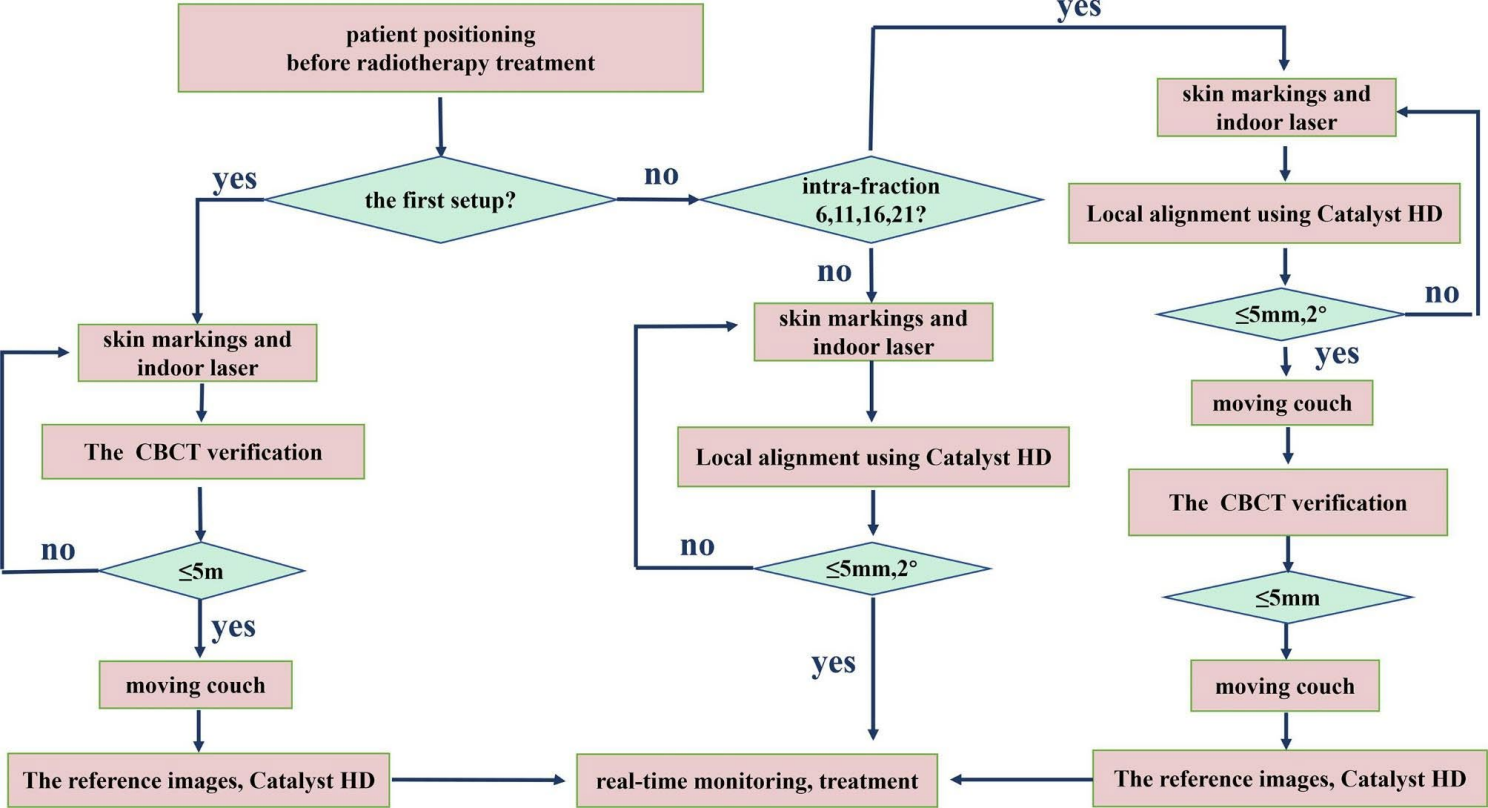
Image guidance workflow Setup errors using CBCT were 3D errors, and the thresholds were 5 mm. Setup errors using SGRT were 6D errors, and the thresholds were 5 mm and 2°

The workflow involved conventional prepositioning (skin markings and indoor lasers), further alignment using Catalyst HD™, and verification through weekly kilovoltage (kV) CBCT imaging (Fig. 1). The established protocol with respect to CBCT scans was as follows: the scanning parameters were 200 mA, 75 kV, and 25 ms for tube current, tube voltage, and scanning time, respectively. If setup errors using CBCT were more than 5 mm in any direction, further CBCT images were taken following repositioning. When the deviations were within 5 mm, they were corrected by moving the couch. These scans were registered to the planning CT using three degrees of freedom and a grey-value algorithm, with the ROI including the ipsilateral breast and spine [12].

For the first fraction, prepositioning was performed, followed by CBCT correction, and then a current surface image was acquired using the Catalyst HD™ as the reference. If it was not the first fraction, a current image was captured by Catalyst HD™ for comparison with the reference, and the deviations were controlled within 5 mm and 2° by manual adjustment. Especially for some special fractions (the first fraction per week, i.e., fractions 6, 11, 16, and 21), SGRT corrections were first performed by moving the couch after the daily SGRT-based adjustment, followed by weekly CBCT verification, and then a current image captured by the Catalyst HD™ was used as a new reference that week. The SGRT scans were registered to the reference using six degrees of freedom (6D) and a non-rigid algorithm, and they were completed using the ROIs of the ipsilateral and whole breasts, respectively [19]. Both ROIs were rectangular, reaching up to the jugular vein incision and down to the lower edge of the bolus, with the whole-breast ROI wrapping around the entire chest wall on both sides and the ipsilateral-breast ROI wrapping around the entire chest wall on one side and the mid-body line on the other.

### Data collection

**Fig. 2:**
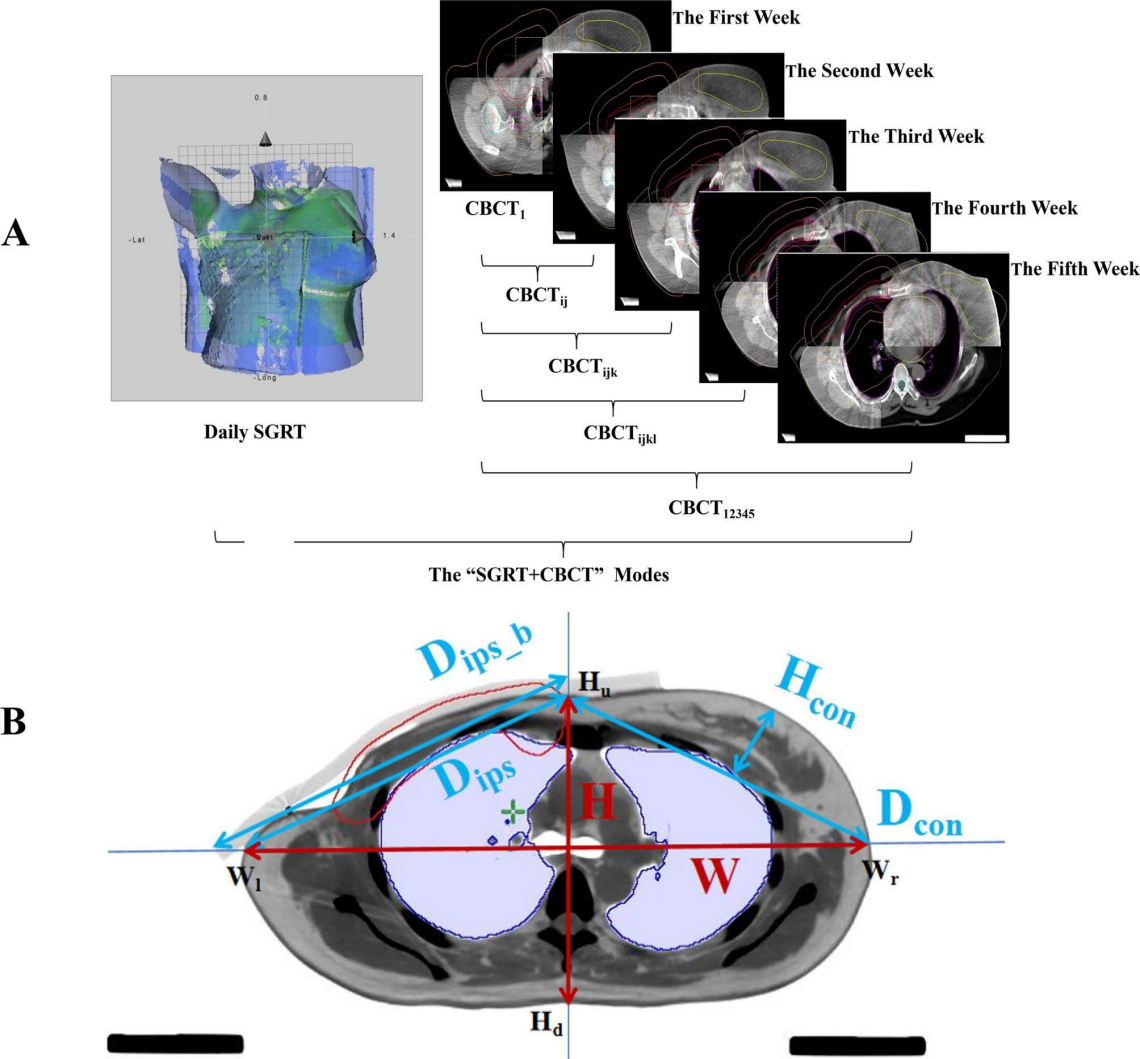
Acquisition of different modes and collection of patient-specific parameters at the bronchial bifurcation level A: Different “SGRT + CBCT” modes. CBCT_ij_, CBCT_ijk_, and CBCT_ijkl_ meant that two, three, four sets of images were randomly selected from five CBCT image sets per patient, respectively. B: Patient-specific parameters at the bronchial bifurcation level

Setup errors were recorded using weekly CBCT and daily SGRT. Based on the scanning frequencies and time intervals, different numbers of CBCT images were randomly selected from each patient’s offline CBCT image atlas and combined with daily SGRT into 16 modes (Fig. 2A).

Furthermore, owing to TPS limitations, 6D SGRT setup errors need to be transformed into 3D deviations in the translational directions based on a previous study [20]. Subsequently, these 3D deviations were imported into the TPS system to recalculate the accumulated doses in the CTVs, PTVs (breast), and OARs (heart, ipsilateral lung, and contralateral breast). A total of 18,400 dose data sets were collected from 25 fractions.

In addition, some patient-specific parameters were measured in the planning CT images (Fig. 2B), and more information on these parameters is shown in Table 1. The diameter and height of the breast have been found to be strongly correlated with its size, and they had the strongest association at the breast centre level (*R* = 0.62 and 0.81, respectively) [21]. Therefore, at the bronchial bifurcation level, including the breast centre (Fig. 2B), we measured the following parameters:

·W: horizontal line across the bronchial bifurcation centre, which was defined as the length from the left point to the right point across the skin surface (W_l_W_r_).

·H: vertical line across the bronchial bifurcation centre, which was defined as the length from the point above to the point below the skin surface (H_u_H_d_).

·D_con_: contralateral breast diameter (H_u_W_r_).

·H_con_: the height of the contralateral breast, was defined as the vertical line of D_con_ across the highest point of the breast skin surface.

·D_ips_: ipsilateral breast diameter (H_u_W_l_).

D_ips_b_: diameter of the ipsilateral breast with a bolus.

·D_ctv_: maximum diameter of the left and right endpoints of the CTV outline.

·T_con_, T, slopes (H_con_/D_con_, H/W, respectively).

Additionally, V_t_ (CTV), age, and BMI of the patients were measured and recorded.

**Table 1:** Patient-specific parameters at the bronchial bifurcation level

patient-specific parameters	Min value	Max value	Median value	Mean value	Standard Deviation
V_t_ (cm^3^)	423.80	634.20	844.70	643.53	102.92
Dctv (cm)	11.61	12.74	15.46	12.86	0.96
Age (year)	30.00	50.00	68.00	50.78	9.67
BMI (kg/m^2^)	20.17	24.65	31.01	25.01	2.94
D_con_ (cm)	17.32	19.52	23.98	19.73	1.62
H_con_ (cm)	3.05	4.45	8.02	4.68	1.10
T_con_	0.16	0.23	0.36	0.24	0.05
D_ips_ (cm)	16.98	19.86	23.91	19.95	1.47
D_ips_b_ (cm)	4.87	21.88	25.82	21.38	3.83
W (cm)	30.14	33.97	39.05	34.41	2.18
H (cm)	16.74	19.03	25.26	19.47	2.10
T	0.49	0.56	0.66	0.57	0.05

### Statistical analyses

Descriptive statistical analyses were performed for setup errors, dosage, and patient-specific parameters. The group mean error (M), systematic error (Σ), and random error (σ) with regard to the transformed 3D deviations of SGRT were calculated, and the CTV–PTV margins were generated based on the van Herk Eq. (2.5Σ + 0.7σ) [22].

The dependence of the ROIs on these modalities was investigated to evaluate the clinical differences, and a paired t-test and non-parametric Wilcoxon matched pairs signed rank test were applied to evaluate if the ROI selections had any impact on the SGRT 3D deviations and dose distribution. The dose differences of the OARs and targets between different modes were assessed using regular ANOVA and Friedman analysis, respectively. Furthermore, the impact of patient-specific parameters on doses in the target volume and OARs were assessed in different modalities, and Spearman and Pearson correlation coefficients were utilised to evaluate dose correlations in all modes, as well as the impact of patient-specific parameters on dosimetry distribution. A significance level of α = 0.05 was established for all statistical studies. All calculations and plotting were performed using *R* programming language and IBM SPSS statistics (version 20, IBM Corporation, Armonk, NY, USA).

## Results

### Setup difference and margins in all modes

All patients underwent clinical tests without incident. After correcting the SGRT-based shifts, the residual setup errors did not exceed 3 mm in any direction. Meanwhile, for all modes, the Shapiro-Wilk tests were used to detect the frequencies of SGRT setup errors in each direction, and they followed a Gaussian distribution.

ROI selection affected the SGRT setup errors in different modes. In the anterior–posterior (AP) direction, compared with the whole breast ROI, the errors with the ipsilateral breast ROI were slightly higher for all modes, except for CBCT_14_, CBCT_134_, and CBCT_1234_ (*P* < 0.05). In the superior–inferior (SI) and left –right (LR)  directions, they did not differ significantly for all modes except for those with CBCT participation at weeks 1, 2, and 3 and a CBCT frequency of > 3. These modes were CBCT_12345_ in LR and CBCT_1234_, CBCT_1235_, and CBCT_12345_ in SI.

As shown in Supplementary Tables 1, for the ipsilateral ROI, the SGRT setup errors and margins tended to decrease as CBCT frequency increased. The decline in Σ was greater than the decline in σ, and contributed more to the decline in margins. For the same frequency, the CBCT time interval had a minimal influence on errors and margins. The margins in all directions exceeded 5 mm when CBCT frequency was less than 3. Compared with the other directions, the margins in SI were the largest for all modes except for CBCT_124_ and CBCT_125_, and they were more than 5 mm, except for CBCT_1245_ (4.97 mm). For the whole breast ROI, the change law was largely uniform (Supplementary Table 2).

### Dose difference of OARs in all modes

**Fig. 3:**
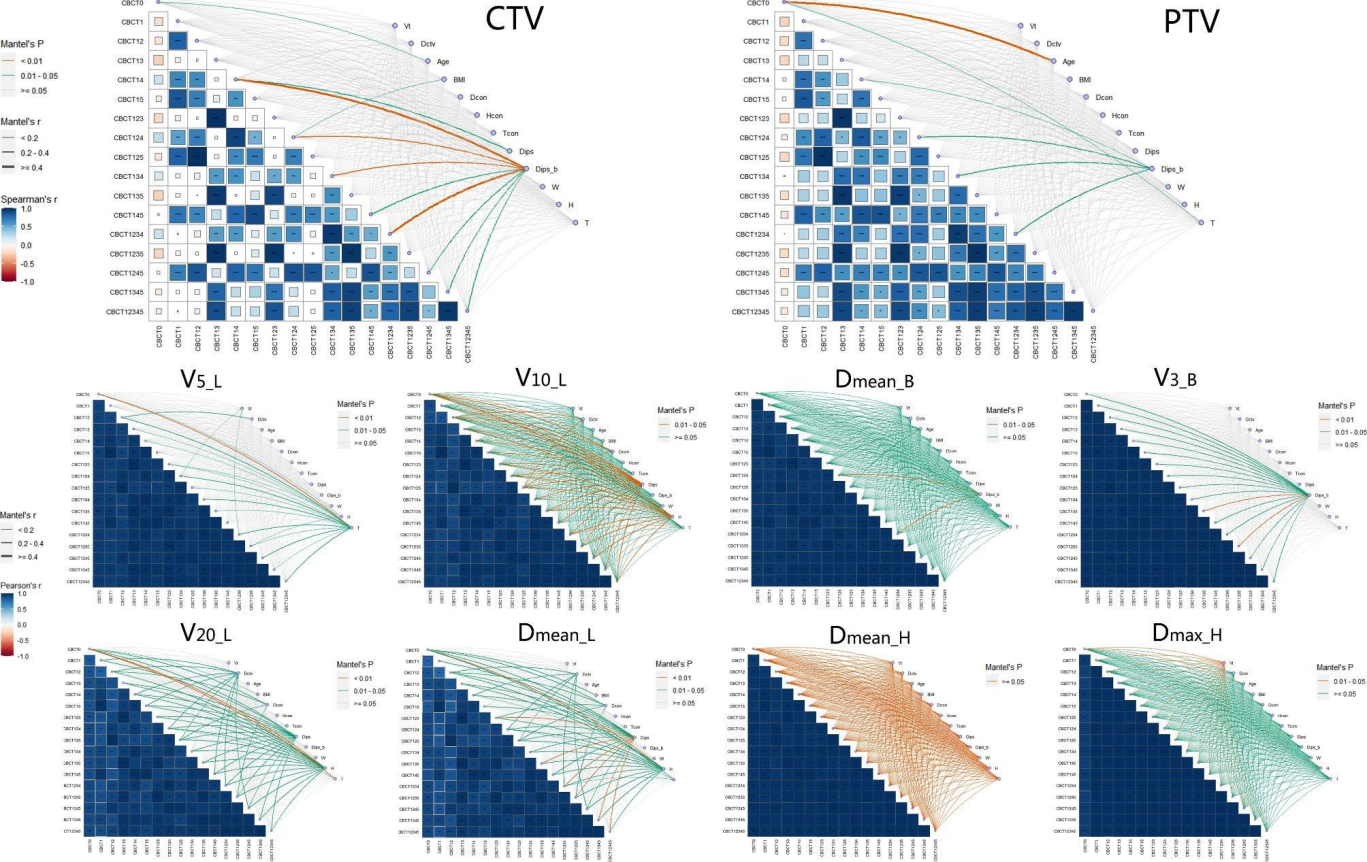
Colour plots of correlation for all modes and patient-specific parameters with the ipsilateral breast ROI

The OAR doses exhibited better correlations than the dose coverage (V95%) of the PTV and CTV between the different modes with the ipsilateral breast ROI (Fig. 3). They showed significant positive correlations with the corresponding theoretical doses (*R* = 0.778–0.990), in which the correlation coefficients in the ipsilateral lung V_20_ and D_mean_ were the lowest (*R* = 0.778–0.884), whereas the others were greater than 0.9. The frequency and time interval of CBCT had little effect on the OAR dose. For all modes, there were strong correlations (*R* > 0.9) in the OAR doses between the different modes, except for the ipsilateral lung’s V_20_ and D_mean_ (*R* = 0.734–0.987).

The patient-specific parameters had the greatest effect on the V_20_ and D_mean_ of the ipsilateral lung but had little effect on the D_mean_ of the heart and contralateral breast. In the V_20_ and D_mean_ of the ipsilateral lung, patients’ D_ctv_, D_con_, D_ips_, and H had some impact on almost all modes (*R* = 0.194 to 0.397). There were moderate correlations between patients’ H and T and ipsilateral lung V_5_ (*R* = 0.170 to 0.270), patients’ D_ips_, H and ipsilateral lung V_10_ (*R* = 0.184 to 0.291), patients’ D_ips_b_ and contralateral breast V_3_ (*R* = 0.248 to 0.391), and patients’ V_t_ and heart D_max_ (*R* = 0.147 to 0.186). The colour plot of the whole breast ROI showed a consistent trend (Supplementary Fig. 1).

There were no significant differences in the OAR doses between the ipsilateral and whole-breast ROIs for all modes, except for CBCT_12_, CBCT_125_, and CBCT_1245_ in the V_3_ and D_mean_ of the contralateral breast (*P* < 0.05). For the same ROI, the OAR doses did not differ significantly between the different modes (*P* > 0.05).

Top: Spearman correlation in dose coverage (V95%) of CTV and PTV among modes and the impact of patient-specific parameters on these doses. Bottom: Pearson correlation of OAR doses (V_5_, V_10_, V_20_, and D_mean_ of ipsilateral lung, V_3_ and D_mean_ of contralateral breast, D_mean_ and D_max_ of heart) among modes and the impact of patient-specific parameters on these doses. The left side of each graph shows the dose correlation between different modes, where blue and red in the squares indicate positive and negative correlations, respectively; the darker the colour, the stronger the correlation. The right side of each graph showed the correlations between patient parameters and doses in different modes, where the line colours represent different *P*-value ranges. Three colours: red represents *P* < 0.01, green represents *P* = 0.01–0.05, and grey represents *P* ≥ 0.05; two colours: red represents *P* = 0.01–0.05, and green represents *P* ≥ 0.05; one colour: red represents *P* ≥ 0.05. The thickness of the lines represents the correlation, three lines: the thinnest, thickest, and the middle-thickness lines represent *R* < 0.2, ≥ 0.4, and 0.2–0.4, respectively; two lines: the thinnest line represents *R* < 0.2, and the thickest line represents *R* = 0.2–0.4.

### Dose difference of V_CTV50Gy_ and V_PTV50Gy_ in all modes

As shown in Fig. 3, for the ipsilateral breast ROI, only V_CTV50Gy_ in CBCT_14_, CBCT_124_, and CBCT_1234_ and V_PTV50Gy_ in CBCT_125_ had moderate correlations with the corresponding theoretical doses, especially in CBCT_124_ (*R* = 0.635). Significant correlations were found between the different modes, accompanied by similar laws for CTV and PTV (*R* = 0.416–0.966). V_CTV50Gy_ and V_PTV50Gy_ in CBCT_12345_ were significantly positively correlated with those in other modes (*R*_CTV_ = 0.455–0.885, *R*_PTV_ = 0.517–0.919), and these correlations showed an upward trend as the CBCT frequency increased.

The V_CTV50Gy_ and V_PTV50Gy_ of these modes with no CBCT participation at week 5 had the highest correlations (*R*_CTV_ = 0.746–0.920, *R*_PTV_ = 0.817–0.966) with those of CBCT only at week 5 on the original basis, except for CBCT_14_, CBCT_124_, and CBCT_134_, which had the highest correlations with CBCT_134_, CBCT_1234_, and CBCT_1234_, respectively (*R*_CTV_ = 0.854, 0.873, and 0.918; *R*_PTV_ = 0.879, 0.912, and 0.890).

The impact of CBCT frequency and time interval on the target doses was significant. For the same CBCT frequency, the correlations between V_CTV50Gy_ and V_PTV50Gy_ increased as the CBCT time interval increased. For instance, the *R*_PTV_ was 0.573, 0.662, 0.668, and 0.820 for CBCT_12_, CBCT_13_, CBCT_14_, and CBCT_15_ in terms of CBCT_1_, and 0.696 and 0.966 for CBCT_134_, CBCT_135_ in terms of CBCT_13_, respectively. For the same CBCT time interval, the correlations first decreased with increasing CBCT frequency, and then increased after adding CBCT at week 5, but the increase in amplitude was not as large as the decline. For instance, the *R*_PTV_ was 0.573, 0.503, 0.436, and 0.565 for CBCT_12_, CBCT_123_, CBCT_1234_, and CBCT_12345_ in terms of CBCT_1_, and 0.695, 0.419, and 0.517 for CBCT_123_, CBCT_1234_, and CBCT_12345_ in terms of CBCT_12_, respectively.

Patient-specific parameters had a prominent influence on CTV and PTV doses. Once CBCT was performed at week 4, the CTV doses of these modes were significantly correlated with D_ips_b_ (*R* = 0.270 to 0.480), while the CTV dose of CBCT_124_ was influenced by patients’ BMI (*R* = 0.198). Only PTV doses of CBCT_14_, CBCT_124_, CBCT_134_, and CBCT_1234_ were affected by D_ips_b_ (*R* = 0.180, 0.256, 0.229, and 0.292, respectively). Patient age was significantly correlated with T (*R* = 0.527), and all of them had an effect on the planned PTV doses (*R* = 0.443 and 0.189, respectively). For the whole-breast ROI, stronger correlations among modes and bigger effects of patient-specific parameters on target doses were found (Supplementary Fig. 1).

Furthermore, there were statistically significant differences in V_CTV50Gy_ and V_PTV50Gy_ between different ROIs, with higher doses in the ipsilateral breast ROI for all modes (*P* < 0.05). For the ipsilateral ROI, the CTV doses among all modes did not differ significantly (*P* > 0.05), and there were small differences in PTV doses only between CBCT_1_ vs. CBCT_1345_ and (CBCT_1_, CBCT_12_, CBCT_14_) vs. CBCT_12345_ (*P* < 0.05).

**Fig. 4:**
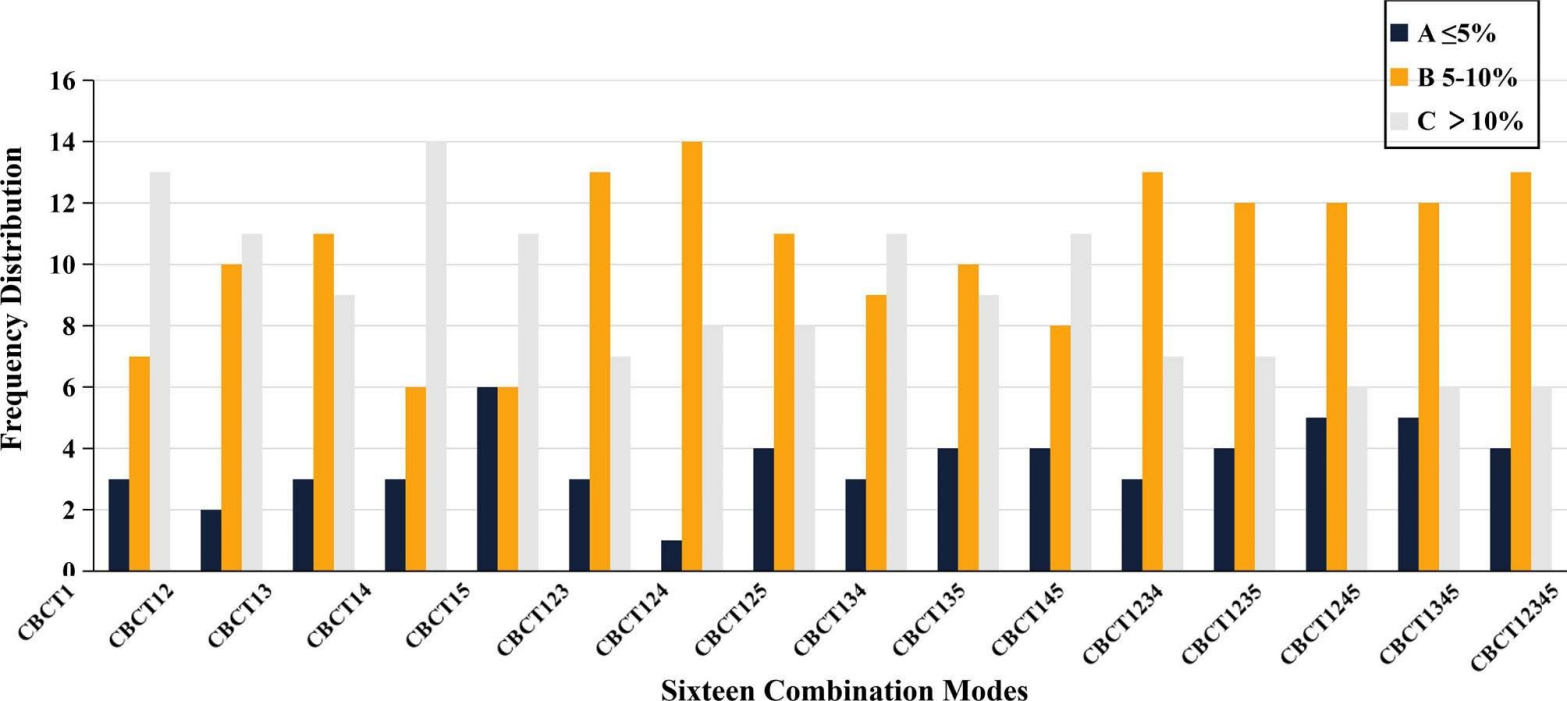
The frequency of PTV deviation percentage in all modes with the ipsilateral breast ROI The frequency with percentage of A, < 5%; B, 5–10%; C, > 10%

Based on quality control experience and early reports [23], the PTV deviation percentage was classified as excellent, good, and unsatisfactory when less than 5%, > 5%, and > 10%, respectively. Only when the CBCT frequency was ≥ 3 (except for CBCT_134_, CBCT_135_, and CBCT_145_), the percentage classified as good was > 65% (Fig. 4). For modes with a CBCT frequency ≥ 4, there was excellent agreement on the determination of the classification (Kappa = 0.715–1), except for CBCT_1234_ (Supplementary Tables 3 and Table 4). High kappa coefficients were achieved between CBCT_124_ and CBCT_1234_, and CBCT_125_, CBCT_1235_ and CBCT_1245_ (Kappa = 0.704–0.901).

## Discussion

The SGRT system with non-ionising and non-invasive optical scanning provides treatment position verification and continuous patient monitoring during RT by providing dynamic surface information. Previous studies on patients with breast cancer who undergo FB have mainly focused on the positioning accuracy of SGRT techniques. MacFarlane et al. reported that the SGRT setup provided dosimetric accuracy similar to that of CBCT by comparing the lung and CTV V_95%_ dose metrics between the CBCT and SGRT setups [15]. Meanwhile, the overall post-CBCT 3D corrections for patients initially aligned with the C-RAD CatalystHD were significantly smaller than those aligned with subcutaneous tattoos [24]. These studies have shown that SGRT is a solid supplemental strategy to CBCT for accurate RT setup, however, little is known about how to combine SGRT and CBCT to guide breast cancer RT more accurately and cost-effectively. In this paper, we describe the clinical introduction of a multiple “SGRT + CBCT” mode fusion, the positioning and dosage differences among these modes, and the optimal strategy for high-precision radiotherapy in patients who underwent mastectomy.

Given that the actual cumulative dose in TPS systems is calculated based only on 3D setup errors, we transformed the original 6D setup errors into 3D errors. There were significant differences in setup errors before and after the changeover, most notably in the LR (0.11 ± 3.58 mm vs. 0.08 ± 3.58 mm for the ipsilateral ROI, 0.10 ± 3.20 mm vs. 0.09 ± 3.21 mm for the whole ROI) and SI (-0.02 ± 3.90 mm vs. -0.03 ± 3.91 mm for the ipsilateral ROI, -0.08 ± 3.51 mm vs. -0.09 ± 3.52 mm for the whole ROI) directions. Previous studies found that rotational errors were not negligible during radiation in patients with breast cancer [19, 25]. Our study’s results were consistent with these results, emphasising the necessity of using modified data again. As a result, it implied that both rotational and translational errors contribute to residual deviations, which should all be corrected for precise radiotherapy.

Similar to other studies, we discovered a significant influence of ROI selection on SGRT-guided radiation [26–28]. Laaksomaa et al. reported that the rotation error using the AlignRT® surface guiding system was lower with a T-shaped ROI than with an O- or B-shape in patients with breast cancer [26]. The B-shaped ROI included the entire breast on the PTV side in the manual outline utilizing the AlignRT. A soft tissue region was excluded from the O-shaped ROI, which was identical to the B-ROI. A T-shaped ROI was created, which included the diaphragm, the sternum, and a tiny piece of breast tissue on both sides. In this work, the Catalyst HD system was utilized to delineate only rectangular and square ROIs, with T-shaped ROIs corresponding to the whole ROI and O- or B-shaped ROIs corresponding to the ipsilateral ROI in terms of range. Based on the converted data, we found that the setup errors using the Catalyst HD system differed only in the AP direction for almost all modes except for CBCT_12345_, and the deviation was greater with the ipsilateral ROI than with the whole ROI. Further analysis revealed that for all modalities, there was essentially no change in the OAR doses when different ROIs were utilised; however, the target doses were considerably variable and higher in the ipsilateral ROI than in the whole ROI. This means that selecting a suitable ROI is crucial during surface image-guided RT, and the ipsilateral ROI could be recommended for patients who underwent mastectomy.

Meanwhile, the frequencies of the SGRT setup errors in all modes followed a Gaussian distribution, such as the frequency distribution of errors in mode CBCT_1_ (Supplementary Fig. 2), which was similar to the motion of a pendulum. Therefore, it was feasible for “SGRT + CBCT” modes to accurately guide positioning for patients with breast cancer receiving postoperative RT in the FB condition based on thresholds of 5 mm and 2°. Supplementary Table 1 shows that the CTV–PTV margins largely depended on the CBCT frequency for all modes with the ipsilateral ROI and decreased with increasing CBCT frequency; however, the CBCT time interval had almost no effect on them. In line with this, some studies reported that the CTV–PTV margins depended on the immobilisation devices, radiation method, and frequency of verification imaging, and a 5–10 mm CTV–PTV margin is required in breast cancer radiotherapy [29, 30]. In this study, we found that the margin in the SI direction was the largest, and only when the CBCT frequency was ≥ 4 could the margins in all directions be approximately 5 mm for patients who underwent supine mastectomy with cushions stuffed with foam pieces. Thus, the oncologists and physicists can adjust the margins appropriately according to the mode of a specific CBCT frequency to improve RT accuracy.

Additionally, we first demonstrated that mode selection had a substantial influence on the target doses but not on the OAR doses, none of which differed among all modes, as shown in Fig. 3. For the CTV and PTV with ipsilateral ROI, the correlations among all modes increased with increasing CBCT time interval, and diminished and subsequently strengthened as the CBCT frequency increased. When CBCT was performed at week 5, these correlations increased dramatically. CBCT_12345_ was significantly positively correlated with all other modes. As a result, the target doses depended on the CBCT frequency and timing, which may be caused by the psychological journey of patients during radiotherapy [31, 32]. Patients had the highest anxiety level before radiotherapy, relaxed after the start of radiotherapy, showed varying degrees of change in mental and psychological status during radiotherapy, and gradually levelled off in the last week, with the lowest anxiety level on the day when the treatment was completed. Hence, we must focus on the selection of the imaging guidance mode based on the patient’s specific situation.

As mentioned above, patient-specific parameters had some impact on dose distribution in various modes (Fig. 3). For the CTV with the ipsilateral ROI, once the modalities had CBCT involved at week 4, their dose distributions were slightly influenced by the patients’ D_ips_b_, with the most influential modalities being CBCT_14_, CBCT_134_, and CBCT_1234_, and CBCT_14_ and CBCT_124_ were dependent on the patient’s BMI. This may be due to a slight change in the patient’s breast volume in the middle and late stages of radiotherapy, resulting in a weak correlation between the patient’s D_ips_b_ and dose in these modes [33, 34]. For the PTV with the ipsilateral ROI, only D_ips_b_ had an effect on the dose (V95%) in modes CBCT_124,_ CBCT_134_, and CBCT_1234_. An interesting phenomenon was that the patients’ age and thoracic slope (T) were positively correlated with the original planned dose, probably because the patients’ age affected their thoracic slope and caused a change in the planned dose. However, these modes without CBCT involvement in week 4, such as CBCT_15_ and CBCT_125_, had dose distributions that were largely unrelated to the differences in patient-specific parameters. This might be related to the limited sample data on breast volume changes in participants included in week 5.

Meanwhile, we found that the relative deviation percentages of the CTV and PTV almost did not differ among all modes with the ipsilateral ROI, and the frequency of their attainment (< 10%) according to the experience and early reports [23] is presented in Fig. 4. Only the frequencies of attainment were high and relatively consistent among CBCT_123_, CBCT_124_, CBCT_125_, and modes with CBCT frequency ≥ 4. Due to the small frequencies of PTV deviation within 5%, we calculated the kappa coefficients between the above modes mainly based on the PTV deviation classification of (≤ 10% and > 10%) and (< 5%, 5-10%, and > 10%). There was great agreement between CBCT_124_ and CBCT_1234_, as well as CBCT_125_, CBCT_ikjl_ and CBCT_12345_. It meant that CBCT_124_ and CBCT_125_ could replace the modalities with a CBCT frequency of ≥ 4 in some ways.

Therefore, the conclusions that were drawn are listed in Table 2. In terms of the cumulative doses and the percentage of PTV deviation, the modes with high consistency were CBCT_123_, CBCT_124_, CBCT_125_, CBCT_iljk_, and CBCT_12345_. In terms of the CTV-PTV margins, they should increase with decreasing CBCT frequency. To improve RT accuracy, oncologists and physicists ought to adjust the margins in accordance with the mode of a certain CBCT frequency. In terms of patient-specific parameters, it was recommended that CBCT be performed at week 4 in order to capture changes in the patient’s anatomical information as early as possible. As a result, further choices for suitable modalities included CBCT_124_, CBCT_1234_, CBCT_1245_, CBCT_1345_, and CBCT_12345_. In terms of the determination of PTV deviation classification, the modalities with the strongest agreement were (CBCT_124_ and CBCT_1234_), (CBCT_125_ and CBCT_1245_), and (CBCT_1245_, CBCT_1345_, and CBCT_12345_).

**Table 2:** The preferred options based on the different dimension and the corresponding margins

Frequency	Recommended mode	Margin(mm)			Dose			
		LR	SI	AP	Deviation	Parameter impact	Agreement(≤10% and > 10%)	Agreement(<5%, 5-10%, and > 10%)
3	CBCT_123_	5.29	6.89	5.38	*		**	**
	CBCT_124_	4.90	5.54	5.72	*	*	***	***
	CBCT_125_	4.79	5.94	6.09	*		****	****
4	CBCT_1234_	4.60	5.61	5.28	*	*	***	***
	CBCT_1235_	4.80	5.94	5.14	*		****, *****	****, *****
	CBCT_1245_	4.55	4.97	5.34	*	*	****, *****	*****
	CBCT_1345_	4.77	5.89	4.89	*	*	*****	*****
5	CBCT_12345_	4.44	5.07	4.94	*	*	*****	*****

Note: Single asterisks in the table represent modes that can be considered initially; multiple asterisks represent modes with an excellent agreement about PTV deviation percentage.

A limitation of our analysis was the absence of an intrafraction SGRT error assessment. However, intrafraction error documentation of patients during treatment has been performed, and this analysis is pending. A further limitation of our study is that although the patient’s respiratory motility was found to be low during treatment monitoring, there was some bias in the respiratory phase between CBCT and SGRT reference acquisition for patients with breast cancer who undergo FB. Meanwhile, owing to the small sample size and lack of daily CBCT, changes in target volume and tumour regression were not significantly explored in this study, which would consequently affect the dependence assessment of patient-specific parameters on the dose distribution in different modalities. Therefore, substantially larger sample sizes and higher CBCT frequencies with DIBH are necessary to assess the optimal fractionation and timing of CBCT and predict the impact of patient-specific parameters on the mode.

## Conclusion

The positioning and dose distribution in RT for patients who underwent mastectomy are affected by image guidance modes and individual patient characteristics. To explore more suitable modes, we proposed the clinical application of 16 “SGRT + CBCT” modes through changing the frequency and time interval of CBCT. SGRT setup errors and CTV–PTV margins were observed among these modes. These modes were recommended with the ipsilateral breast as the ROI and a combination of daily SGRT and a CBCT frequency of ≥ 3, and CBCT must be included in the first and second weeks when the frequency was 3. Considering the additional radiation and time consumption of CBCT, as well as the early acquisition of varying anatomical information from patients, CBCT_124_ can be prioritized. If it cannot be done at week 4 owing to treatment stoppage, equipment malfunction, or other circumstances, it was suggested that CBCT must be done at week 5. Therefore, it is possible to precisely manage tumour motion using modalities of daily SGRT combined with less frequent CBCT.

## Electronic supplementary material

Below is the link to the electronic supplementary material.

Supplementary Material 1

## Data Availability

The data that support this study are not openly available due to ethical and privacy concerns and are available from the corresponding author upon reasonable request.

## List of Abbreviations

RT: Radiation therapy
IGRT: Image-guided radiation therapy
CBCT: Cone-beam computed tomography
SGRT: Surface-guided radiotherapy
FB: Free-breathing
DIBH: Deep-inspiration breath-holding
BMI: Body mass index
OARs: Organs at risk
ROIs: Regions of interest
TPS: Treatment planning system
CTV: Clinical target volume
RTOG: Radiation Therapy Oncology Group
PTV: Plan target volume
CW: The chest wall
IMNs: Internal lymph mammary nodes
IMRT: Intensity-modulated radiotherapy
W: Horizontal line across the bronchial bifurcation centre, which was defined as the length from the left point to the right point across the skin surface
H: Vertical line across the bronchial bifurcation centre, which was defined as the length from the point above to the point below the skin surface
Dcon: Contralateral breast diameter at the bronchial bifurcation level
Hcon: The height of the contralateral breast, was defined as the vertical line of Dcon across the highest point of the breast skin surface
Dips: Ipsilateral breast diameter at the bronchial bifurcation level
Dips_b: Diameter of the ipsilateral breast with a bolus at the bronchial bifurcation level
Dctv: Maximum diameter of the left and right endpoints of the CTV outline at the bronchial bifurcation level
Tcon, T: Slopes (Hcon/Dcon, H/W, respectively)
M: Group mean error
Σ: Systematic error
σ: Random error
AP: anterior–posterior
LR: left–right
SI: superior–inferior
