# Supplementary material for: Optimal fractionation and timing of weekly cone-beam CT in daily surface-guided radiotherapy for breast cancer
Source: Radiat Oncol. 2023 Jul 5;18:112. doi: 10.1186/s13014-023-02279-4 (PMC10320897; doi:10.1186/s13014-023-02279-4)
Supplement: Supplementary file 1 — Supplementary Material 1 [file 13014_2023_2279_MOESM1_ESM.docx]

Supplementary table 1. SGRT setup errors in all modes with the ipsilateral ROI and the corresponding margins (mm)

|  |  | **LR** | | | | **SI** | | | | **AP** | | | |
| --- | --- | --- | --- | --- | --- | --- | --- | --- | --- | --- | --- | --- | --- |
| **frequency** | **modes** | **M** | **Σ** | **σ** | **Margin** | **M** | **Σ** | **σ** | **Margin** | **M** | **Σ** | **σ** | **Margin** |
| 1 | CBCT_1_ | 0.07 | 1.80 | 3.43 | 6.91 | -0.03 | 2.47 | 3.23 | 8.45 | 0.67 | 1.85 | 2.73 | 6.53 |
| 2 | CBCT_12_ | 0.10 | 1.22 | 3.23 | 5.30 | -0.03 | 1.94 | 3.49 | 7.30 | 0.68 | 2.05 | 2.98 | 7.21 |
|  | CBCT_13_ | -0.06 | 1.29 | 3.31 | 5.55 | -0.04 | 2.18 | 3.21 | 7.71 | 0.86 | 1.40 | 2.60 | 5.32 |
|  | CBCT_14_ | 0.07 | 1.36 | 3.36 | 5.76 | -0.07 | 1.63 | 3.32 | 6.40 | 0.70 | 1.49 | 2.82 | 5.69 |
|  | CBCT_15_ | 0.02 | 1.48 | 3.29 | 5.99 | -0.11 | 2.00 | 3.20 | 7.25 | 0.74 | 1.51 | 2.75 | 5.70 |
| 3 | CBCT_123_ | 0.02 | 1.25 | 3.09 | 5.29 | -0.06 | 1.92 | 2.98 | 6.89 | 0.89 | 1.45 | 2.52 | 5.38 |
|  | CBCT_124_ | 0.12 | 1.08 | 3.16 | 4.90 | -0.16 | 1.36 | 3.04 | 5.54 | 0.71 | 1.49 | 2.85 | 5.72 |
|  | CBCT_125_ | 0.02 | 1.07 | 3.02 | 4.79 | -0.18 | 1.49 | 3.18 | 5.94 | 0.74 | 1.64 | 2.85 | 6.09 |
|  | CBCT_134_ | 0.02 | 1.08 | 3.22 | 4.96 | -0.12 | 1.61 | 3.24 | 6.30 | 0.77 | 1.35 | 2.72 | 5.29 |
|  | CBCT_135_ | -0.06 | 1.15 | 3.17 | 5.09 | -0.12 | 1.82 | 3.15 | 6.76 | 0.85 | 1.29 | 2.65 | 5.08 |
|  | CBCT_145_ | 0.01 | 1.26 | 3.20 | 5.39 | -0.15 | 1.55 | 3.15 | 6.07 | 0.78 | 1.33 | 2.73 | 5.24 |
| 4 | CBCT_1234_ | 0.10 | 1.00 | 3.00 | 4.60 | -0.14 | 1.43 | 2.92 | 5.61 | 0.80 | 1.37 | 2.66 | 5.28 |
|  | CBCT_1235_ | 0.02 | 1.10 | 2.94 | 4.80 | -0.15 | 1.57 | 2.86 | 5.94 | 0.88 | 1.34 | 2.57 | 5.14 |
|  | CBCT_1245_ | 0.07 | 0.99 | 2.98 | 4.55 | -0.24 | 1.18 | 2.88 | 4.97 | 0.79 | 1.37 | 2.74 | 5.34 |
|  | CBCT_1345_ | -0.03 | 1.06 | 3.01 | 4.77 | -0.20 | 1.50 | 3.08 | 5.89 | 0.85 | 1.23 | 2.59 | 4.89 |
| 5 | CBCT_12345_ | 0.05 | 1.00 | 2.77 | 4.44 | -0.23 | 1.26 | 2.75 | 5.07 | 0.88 | 1.27 | 2.52 | 4.94 |

Supplementary table 2. SGRT setup errors in all modes with the whole ROI and the corresponding margins (mm)

|  |  | **LR** | | | | **SI** | | | | **AP** | | | |
| --- | --- | --- | --- | --- | --- | --- | --- | --- | --- | --- | --- | --- | --- |
| **frequency** | **modes** | **M** | **Σ** | **σ** | **Margin** | **M** | **Σ** | **σ** | **Margin** | **M** | **Σ** | **σ** | **Margin** |
| 1 | CBCT_1_ | 0.10 | 1.80 | 3.21 | 6.75 | 0.05 | 2.25 | 2.99 | 7.73 | 0.38 | 1.59 | 2.64 | 5.81 |
| 2 | CBCT_12_ | 0.06 | 1.02 | 2.86 | 4.56 | -0.02 | 1.65 | 2.94 | 6.17 | 0.29 | 1.44 | 2.59 | 5.41 |
|  | CBCT_13_ | -0.07 | 0.99 | 2.90 | 4.49 | -0.04 | 1.79 | 2.98 | 6.56 | 0.63 | 1.18 | 2.52 | 4.71 |
|  | CBCT_14_ | 0.10 | 1.33 | 3.05 | 5.46 | -0.22 | 1.33 | 3.16 | 5.54 | 0.52 | 1.39 | 2.73 | 5.37 |
|  | CBCT_15_ | 0.10 | 1.47 | 2.98 | 5.76 | -0.22 | 1.83 | 2.80 | 6.53 | 0.55 | 1.28 | 2.61 | 5.04 |
| 3 | CBCT_123_ | 0.03 | 0.87 | 2.61 | 4.01 | -0.09 | 1.56 | 2.72 | 5.81 | 0.64 | 1.18 | 2.39 | 4.61 |
|  | CBCT_124_ | 0.17 | 0.91 | 2.74 | 4.20 | -0.27 | 1.18 | 2.94 | 5.02 | 0.50 | 1.29 | 2.60 | 5.05 |
|  | CBCT_125_ | 0.12 | 0.99 | 2.58 | 4.29 | -0.28 | 1.32 | 2.63 | 5.15 | 0.48 | 1.20 | 2.49 | 4.74 |
|  | CBCT_134_ | 0.08 | 0.97 | 2.80 | 4.38 | -0.26 | 1.30 | 3.02 | 5.37 | 0.63 | 1.30 | 2.57 | 5.05 |
|  | CBCT_135_ | 0.03 | 1.06 | 2.64 | 4.51 | -0.26 | 1.47 | 2.78 | 5.63 | 0.70 | 1.11 | 2.45 | 4.50 |
|  | CBCT_145_ | 0.10 | 1.26 | 2.86 | 5.16 | -0.27 | 1.35 | 2.73 | 5.28 | 0.61 | 1.19 | 2.57 | 4.78 |
| 4 | CBCT_1234_ | 0.18 | 0.76 | 2.53 | 3.68 | -0.31 | 1.16 | 2.68 | 4.76 | 0.64 | 1.27 | 2.46 | 4.88 |
|  | CBCT_1235_ | 0.13 | 0.95 | 2.32 | 4.01 | -0.31 | 1.24 | 2.46 | 4.82 | 0.72 | 1.11 | 2.31 | 4.40 |
|  | CBCT_1245_ | 0.16 | 0.90 | 2.50 | 4.00 | -0.33 | 1.08 | 2.53 | 4.48 | 0.58 | 1.13 | 2.42 | 4.52 |
|  | CBCT_1345_ | 0.08 | 1.00 | 2.54 | 4.28 | -0.32 | 1.22 | 2.61 | 4.87 | 0.71 | 1.15 | 2.38 | 4.53 |
| 5 | CBCT_12345_ | 0.18 | 0.85 | 2.22 | 3.68 | -0.36 | 1.01 | 2.23 | 4.10 | 0.72 | 1.13 | 2.24 | 4.39 |


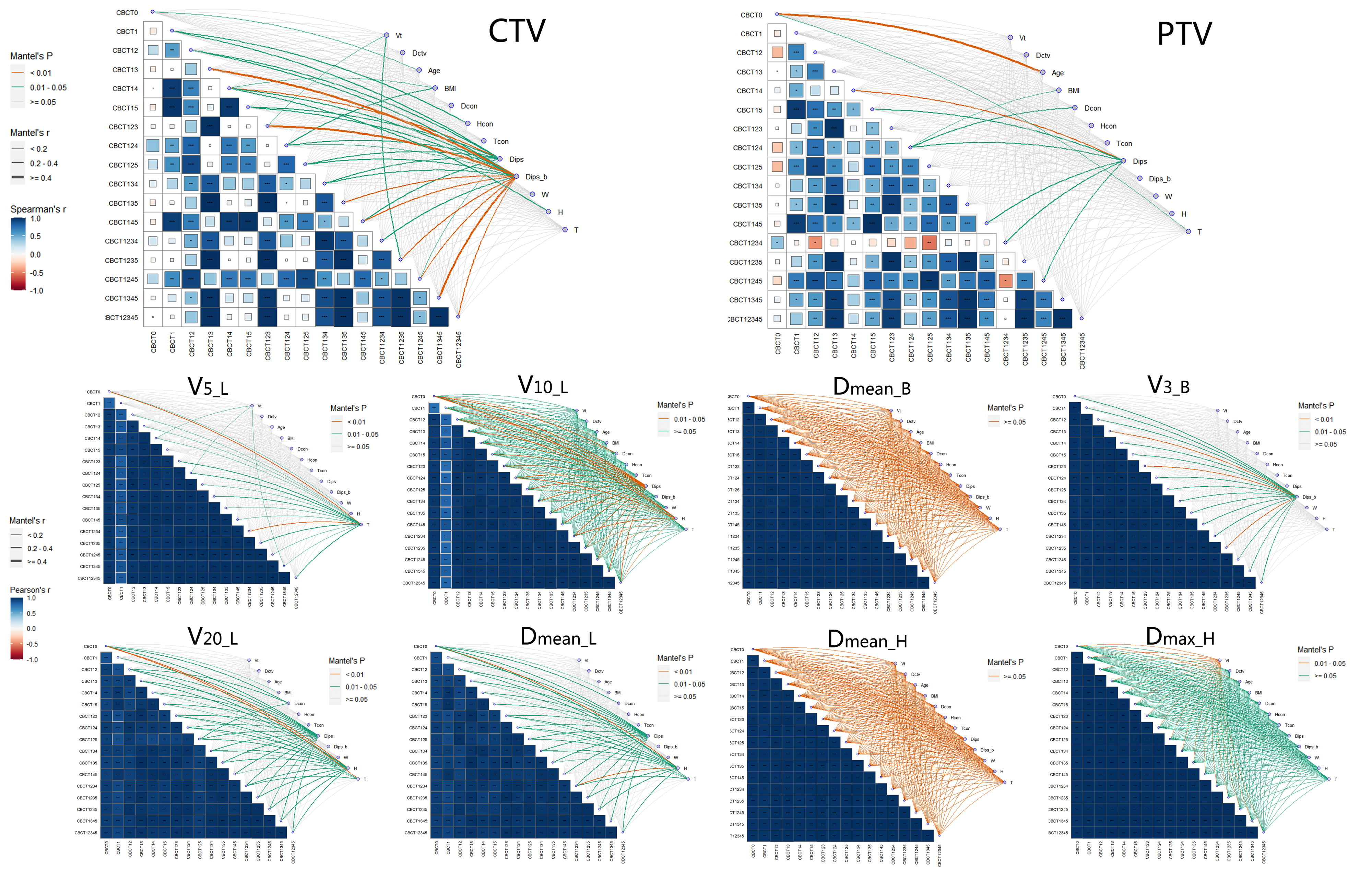


Supplementary Fig. 1 Colour plots of the Spearman or Pearson correlation coefficients for all modes and individualized parameters of patients in the CTV, PTV and OAR with the whole ROI

Supplementary table 3 The Kappas analysis among the most compliance modes with the ipsilateral ROI (≤10% and >10%)

|  | CBCT_123_ | CBCT_124_ | CBCT_125_ | CBCT_1234_ | CBCT_1235_ | CBCT_1245_ | CBCT_1345_ | CBCT_12345_ |
| --- | --- | --- | --- | --- | --- | --- | --- | --- |
| CBCT_123_ | 1 |  |  |  |  |  |  |  |
| CBCT_124_ | 0.506 | 1 |  |  |  |  |  |  |
| CBCT_125_ | 0.506 | 0.425 | 1 |  |  |  |  |  |
| CBCT_1234_ | 0.384 | 0.901 | 0.309 | 1 |  |  |  |  |
| CBCT_1235_ | 0.589 | 0.506 | 0.704 | 0.384 | 1 |  |  |  |
| CBCT_1245_ | 0.465 | 0.593 | 0.796 | 0.465 | 0.893 | 1 |  |  |
| CBCT_1345_ | 0.465 | 0.389 | 0.593 | 0.465 | 0.893 | 0.775 | 1 |  |
| CBCT_12345_ | 0.465 | 0.389 | 0.593 | 0.465 | 0.893 | 0.881 | 1 | 1 |

Supplementary table 4 The Kappas analysis among the most compliance modes with the ipsilateral ROI

(<5%, 5%-10%, and >10%)

|  | CBCT_123_ | CBCT_124_ | CBCT_125_ | CBCT_1234_ | CBCT_1235_ | CBCT_1245_ | CBCT_1345_ | CBCT_12345_ |
| --- | --- | --- | --- | --- | --- | --- | --- | --- |
| CBCT_123_ | 1 |  |  |  |  |  |  |  |
| CBCT_124_ | 0.537 | 1 |  |  |  |  |  |  |
| CBCT_125_ | 0.421 | 0.341 | 1 |  |  |  |  |  |
| CBCT_1234_ | 0.543 | 0.846 | 0.349 | 1 |  |  |  |  |
| CBCT_1235_ | 0.631 | 0.553 | 0.646 | 0.558 | 1 |  |  |  |
| CBCT_1245_ | 0.491 | 0.489 | 0.720 | 0.491 | 0.715 | 1 |  |  |
| CBCT_1345_ | 0.491 | 0.343 | 0.511 | 0.491 | 0.715 | 0.716 | 1 |  |
| CBCT_12345_ | 0.549 | 0.395 | 0.500 | 0.549 | 0.781 | 0.782 | 0.782 | 1 |


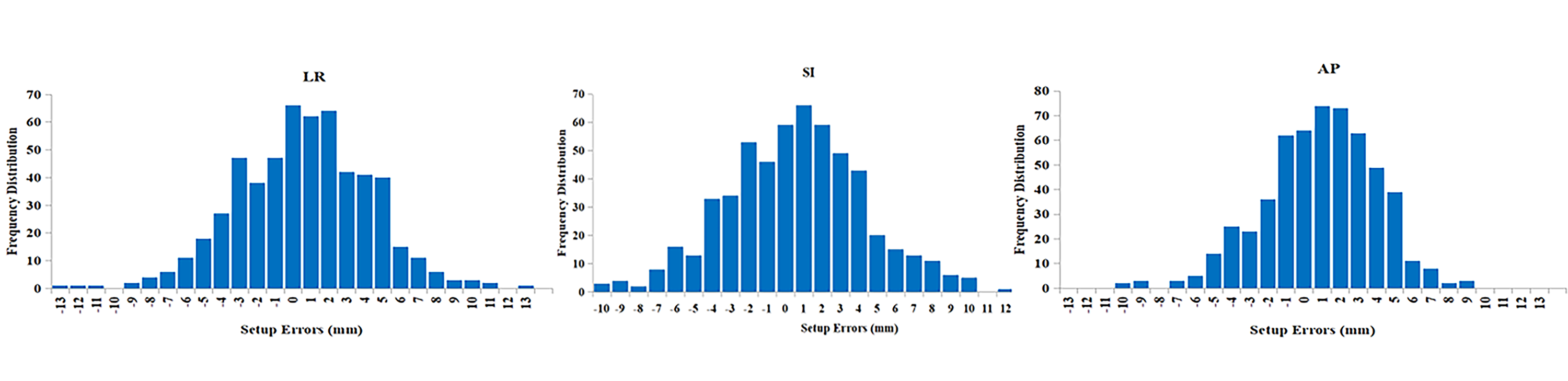


Supplementary Fig. 2 The frequency distribution of errors in mode CBCT_1_
